# Understanding and Preventing Attacks on Health Facilities During Armed Conflict in Syria

**DOI:** 10.2147/RMHP.S237256

**Published:** 2020-03-18

**Authors:** Abdulaziz Omar

**Affiliations:** 1 Institute for Global Health, University College London, London, UK

**Keywords:** Syria, health attacks, international humanitarian law, health workers, health professionals

## Abstract

**Background:**

Despite healthcare facilities being deemed untouchable in times of conflict, the war in Syria has seen its government as well as opposition forces, target their people and infrastructure as a strategy of war. Violations of medical neutrality and International Humanitarian Law has led to the loss of countless medical personnel, civilians and health care facilities; setting the country back to health levels last seen thirty years ago. It is evident through the strategy of the Syrian and Russian government that healthcare facilities are being deliberately targeted with humanitarian organisations condemning all parties involved for violating the Geneva Conventions. The report examines the impact of the conflict in Syria on its health facilities and looks at the reasons why these services are under attack and the international response to the conflict. The report concludes by looking into plans currently implemented to protect our healthcare infrastructure during times of war whilst comparing it to past strategies.

**Methods:**

A literature review was conducted for the study with information and data collected through several search engines including Google Scholar, PubMed, MEDLINE, OVID and searches through Google. The keywords mapped to find relevant literature includes “Syria”, “healthcare”, “health care worker”, “humanitarian aid”, and “volunteer”, “International Humanitarian Law”, “Geneva Convention”. The majority of the data used was adapted from Physicians for Human Rights (PHR) and the World Health Organisation (WHO). Limitations included using sources written in English due to limited resources to translate literature in Arabic.

**Results:**

The conflict in Syria and deliberate targeting of healthcare facilities has left services decimated with an estimated 782 medical personnel killed during this time; doctors accounting for 32% of total deaths in the five years. Several facilities are also operating at 1% or less functionality.

**Conclusion:**

The results and review highlight the need for protection of health facilities from humanitarian violations as health care continues to be targeted as a strategy of war. The number of attacks has steadily remained constant throughout the years and nothing seems to be done in bringing perpetrators to justice for violations of International Humanitarian Law. The paper calls for more public attention to shed light on the atrocities being committed and further inquiries like the preliminary carried out by *The Lancet* – American University of Beirut.

## Introduction

Nine years on from the start of the Syrian war, the conflict has been and remains to be at the forefront of international debate and discussion.^1^ With much light being shed on the ever-growing refugee crisis and the devastating impact of the crossfire on civilian life, not much has been said about a large factor essential to rebuilding the country: healthcare provision. The complete collapse of Syria’s public health system has been less well documented in mainstream media as pressure on its services continue to be strained as Syria withstands the largest humanitarian crisis in the world currently.^2^ In this report, we examine the impact that the conflict in Syria has had on its healthcare services whilst trying to understand why the sector is under attack and inspecting the plans in place in preventing such attacks in the future. We will also explore the key stakeholders involved in the international response to the situation providing background and context to each one. The report will conclude by looking into plans currently implemented to protect our healthcare infrastructure during times of war whilst comparing it to past strategies and analysing their efficacy and policies moving forward.

## The Health Crisis

### Humanitarian Law

Since the dawn of time, humans have been waging war as a means of settling disputes and disagreements. War not only devastates human lives, buildings and possessions, it destroys the fabric of society and hampers a nation’s infrastructure as everything collapses; civilians often taking the brunt of the hardship. Genocide, plunder, rape and violence of every kind are the insatiable truths of war. Despite this, war is widely recognised and accepted to have limits. Human conflict has been a reality since early civilization and with that, limits stem from the behaviours and customs of wars from history as well as establishing roots from cultural and religious teachings. In the twelfth century, it was ethical, religious and cultural ideologies that led Sultan Saladin to order treatment of not only his wounded army to be tended to in a camp just outside of Jerusalem, but also demanded that his wounded adversaries – the crusaders – also be protected and treated.^3^ The foundations of modern humanitarian law are built on these ethical principles and were later cemented as legislation in 1864 under the initiation of Henry Dunant: the first recipient of the Nobel Peace Prize.^4^

#### The Geneva Conventions

Dunant, who had initially been primarily known as a Swiss businessman, had embarked on a business trip to Northern Italy when he had observed over 40,000 wounded, dying and dead soldiers and civilians at the Battle of Solferino.^5^ Overwhelmed by the sheer number of casualties and the lack of available supplies to care for the wounded, Dunant went on to set up improvised hospitals and organised assistance for the care of the sick.^6^ He later went on to document his experiences in Solferino in his book “Un Souvenir de Solférino”. It is in this same very book where he postulated the idea of there being a neutral body or organisation in place to protect wounded soldiers who not partaking in the hostility of combat.

The release of Dunant’s book led to widespread international deliberation and in 1863, the formation of a commission discussing much of the recommendations from Dunant’s experience.^7^ This committee is considered to be the origin for the creation of the International Committee of the Red Cross. One year on from their first meeting, an international conference was conducted with governments from all European countries as well as the United States, Mexico and Brazil invited.^8^ It was at this diplomatic conference that the first of the four Geneva conventions were introduced in 1864.

The first Geneva Convention was established in to protect all personnel deemed either wounded and sick without bias, regardless of allegiance.^9^ The treaty also saw the introduction of medical neutrality where healthcare workers and medical provisions on the ground would be free from attack during times of war.^10^

The Geneva Conventions would be amended and extended between 1864 and 1949 with two additional protocols finally being added in 1977. The Geneva Conventions are described as the following. The first Geneva Convention protects wounded and sick military personnel on the ground and introduces neutrality to medical and religious persons. The second Geneva convention replaced the Hague Convention of 1907 and is an extension of the first treaty, ensuring that war victims at sea are also protected as well as the protection of medical assistance and provision such as hospital ships.^11^ The third convention was introduced in 1929 and stipulates the treatment of persons deemed to be a prisoner of war requiring humane treatment of all captured.^9^ All the Geneva Conventions up to 1949 had concerned the treatment of those involved directly in the conflict, namely soldiers and the relevant medical professionals. The adoption of the Fourth Geneva Convention in 1949 specifically concerned the protection of civilians during armed conflict. It specifies the responsibility that the party in control of a region has in protecting its civilians as well as humanitarian provision for the population. These four principles have been signed and ratified by all States and are applicable worldwide. These principles form a basis of international humanitarian law and form a custom that all states and countries, whether they have ratified the agreement or not, must endeavour to conduct wartime under these conditions for the good of civilian, humanitarian and military personnel.^12^

## Syrian Conflict

With the conflict now in its eighth year, the number of refugees has soared to 5.6 million with a further 6.6 million Syrians internally displaced in what is now the largest refugee crisis since the Second World War.^13^ One of the biggest impacts in Syria’s rebuilding process has been the constant strain the public health system has been under, not only due to the increasing number of casualties coupled with health workers feeling the country, but facilities have also come under attack as the “weaponisation” of the health continues to be rife.^14^

**Figure 1:**
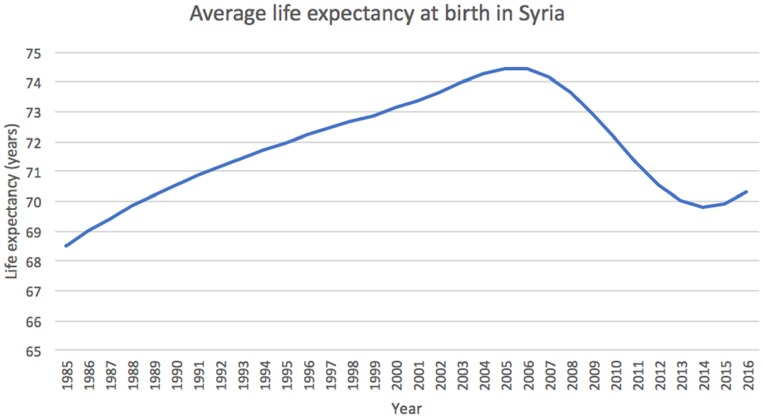
The chart shows how Syria’s average life expectancy reached a low of 69.8 years in 2014 at the height of the conflict. It was last recorded at 70.3 in 2016 - a similar expectancy seen 29 years prior. Data adapted from the World Bank.^16^

By the end of 2017, it was estimated that more than 50% of the country’s hospitals and clinics were deemed either partially open or not fit enough for practice; whether that be due to lack of services or irreparable damage.^15^ The impact of the conflict has seen the largest indicator of health – average life expectancy at birth – set back considerably as seen in Figure 1.^16^ Before the war, Syria had made considerable strides in improving the nation’s overall health with average life expectancy rising from 52 years in 1960 to its peak of 74.4 years in 2006. Since then, figures have dropped drastically with a low of 69.8 years seen in 2014, a figure last seen in 1989.^16^

### Impact of Conflict on Healthcare

One of the most devastating tragedies during the eight-year conflict has been the demise of the country’s health system. The acute destruction of the country has overshadowed the chronic issues Syria stands to face long after the war’s end. The interruption of regular primary health services including but not limited to the caring for women and children; chemotherapy and radiotherapy treatment for patients with cancer and dialysis treatment stands to increase the country’s mortality rate as a consequence despite the deaths being preventable and treatable. Many have been dissuaded by attending their local clinics with the knowledge that what was once a beacon of hope and respite had now become a target; others, unable to make the journey to their closest centres.

The internal displacement of the Syrian people has forced them to seek refuge in regions and camps which would be deemed safer than their homes. The increasing population density has overwhelmed the supply of water and food leading to poor sanitation in these regions. Naturally, this had led to an outbreak of disease amongst the population with an outbreak of measles being reported by WHO nationwide in 2017.^17^,^18^ Under the aid of several humanitarian organisations such as WHO and UNICEF, were able to establish a nationwide campaign encouraging emergency vaccination which was able to aid 4.8 million children across Syria.^17^ A further 2.8 million were vaccinated against polio which included 144,00 children in Raqqa – a city which had been under ISL control from January 2014 to October 2017.^19^ Nevertheless, the rebuilding process cannot begin as long as the health system is under constant attack whilst civilians continue to bear the brunt of the burden.

The World Health Organisation (WHO) defines an “attack” as

any act of verbal or physical violence that obstructs or threatens to interfere with the availability and delivery of health care services during emergencies, and/or with patients’ access to health care.^17^

**Figure 2:**
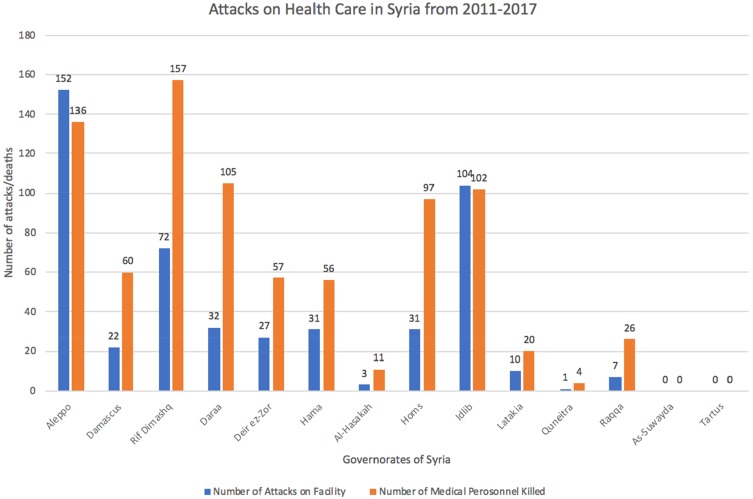
The chart shows the number of healthcare workers that have been killed, from March 2011 to December 2017, across all of Syria’s governorates. It also shows the number of healthcare facilities which have been attacked during the same period. Data from Physicians for Human Rights. *A Map of Attacks on Health Care in Syria*. Available at: https://syriamap.phr.org/#/en. Accessed 2 March 2020.

Organisations such as Physicians for Human Rights (PHR) advocate for attacks against health workers by bringing atrocities and humanitarian law violations to light using medical and scientific bases. According to PHR, between the start of the conflict and December 2017, there has been a total of 492 attacks on medical facilities with 91% of the attack being attributed to the Syrian government directly or its allies.^20^ These attacks were carried out on 330 separate medical facilities throughout the country, with Aleppo’s provisions having been hit the most as seen in Figure 2.^21^

**Figure 3:**
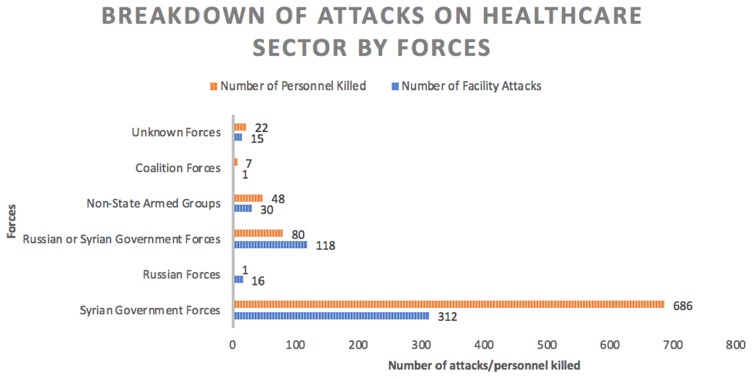
The chart shows the number of attacks on health care facilities as well as the number of personnel killed; this is plotted against the party responsible. Syrian government forces and its allies were responsible for 492 attacks on facilities between 2011 and 2017. Non-state armed groups refer to ISIS and its opposition. Adapted from data collected by the PHR between 2011 and 2017.

Coupled with the destruction of Syria’s health facilities, the loss of life is at a much graver cost to the nation; some argue that the loss of life had been primarily at the hands of the Syrian government and its allies due to the number of attacks on its facilities since 2011. Figure 3 shows that since 2011, the Syrian government has solely been responsible for 312 attacks on health care facilities with the majority of these attacks being chosen and targeted specifically - a direct violation of international humanitarian law.^21^ The intention of directly attacking hospitals is made clear by the nature of the attacks: the same hospitals were targeted multiple times on 79 separate occasions such as Aleppo’s Dar al Shifa Hospital, Azaz National Hospital and the Central Cave Hospital in northern Hama which has been hit 11 times; a further 59 different facilities, which were situated in remote settings without a dense populace or number of buildings, had been attacked pointing to its direct targeting.^21^,^22^

#### Practice in Besieged Areas

The state of medical provisions in opposition-controlled areas are deemed to be well below standard relative to those in regions under government control. With over 1 million citizens currently under siege, this has had a profound effect on the availability of services including medical stores and staff.^23^ Again, the government has been accused of preventing the supply of humanitarian aid as well as medicine and provision of staff. An appeal to the government in 2016 from the Syrian American Medical Society (SAMS) advocated for the relief of the people of Aleppo where 400,000 citizens were left trapped following a bombardment of attacks leaving all hospitals in Eastern Aleppo as either closed or partially open.^24^ Health workers are being forced into practising with makeshift equipment in unsanitary conditions fitting for widespread outbreak in an area under siege; partially since the government have refused to address basic public health by not restoring water chlorination services nor providing vaccines against an outbreak.^25^ In 2018, the region of Eastern Ghouta has suffered a parallel fate to that of Eastern Aleppo in 2016. According to the UN Office for the Co-ordination of Humanitarian Affairs, Eastern Ghouta accounted for 95% of Syria’s 419,920 citizens under siege.^26^ Whilst a large majority of those who have died during the conflict was directly due to the war, approximately 70,000 have died due to the lack of public health whether that be lack of medicine, food, water, sanitary living conditions or vaccination.^27^

## Weaponisation of Healthcare

The “weaponisation of healthcare” in Syria is a term that has recently been brought to the forefront following an inquiry into the conflict by the American University of Beirut.^14^ The paper looks into the repeated violations of international humanitarian law committed through the targeting of healthcare workers and facilities. The difficulties that healthcare workers are under and how they differ from region to region which has led to their roles developing to meet demands are also discussed. Weaponisation alludes to a plethora of facets which has been prevalent and instrumental to the Syrian conflict; often playing a vital role in the strategy of this war.

### Strategy of War

It is evident that attacks on healthcare have played an important role in the war so far. A practice known as a “double-tap attack” had been a technique used by the Central Intelligence Agency (CIA) in their bombardment of the Taliban in Pakistan in 2012.^28^ This tactic involves initiating strikes at a specific location and target followed by a secondary attack, targeting groups assisting the wounded and dead. This is a direct violation of the Geneva Conventions since these attacks target the wounded and health care workers tending to them who are both deemed hors de combat. Volunteer organisations like the Syrian Civil Defence, more commonly referred to as the White Helmets, provide relief to the wounded in response to attacks. Despite their aim of providing relief and rescue in times of crisis, they were caught in 149 double-tap attacks in 2016 alone with 154 workers being killed between 2013 and 2016.^14^

However, these attacks on facilities could be seen as necessary due to the unfortunate circumstance of these facilities becoming strategical pieces in war. At the start of the conflict, emergency humanitarian and healthcare provisions would often be erected away from known military operations to reduce the amount of firefight and conflict that surrounded them. Now, armed forces see hospitals as strongholds to seek refuge in and store weapons, all under the premise that the hospitals are protected under the Geneva Conventions; deterring or at least reducing any chance of an attack. As a result, seeking prosecution against these attacks is often extremely difficult since clear intent and motive must be established for the attack – which is challenging to prove – before the war crime could be tried at the International Criminal Court (ICC).^29^ The fact that Syria has not ratified the treaty on which the ICC is based on, they cannot be prosecuted for any potential war crime under ICC jurisdiction. To be tried at the ICC, Syria must be referred by the United Nations Security Council, something that has only happened only twice in its history: Darfur, Sudan in 2005 and Libya in 2011.^30^,^31^

### Medical Neutrality

The Hippocratic Oath forms the earliest basis of medical ethics and states the basic principles that doctors should strive towards. The oath states “Into whatsoever houses I enter, I will enter to help the sick”; this has been interpreted as doctors have a duty to help those in need, regardless of whatever “house” they may be from and without bias or judgement.^32^ In times of armed conflict, this ethical principle is protected by what is known as medical neutrality. Health care workers must be allowed to carry out their duties in tending to the wounded and sick working in their best interest, regardless of what side they may be on. Medical neutrality allows for the treatment of all those in need without interference. Any dismissal of this principle is deemed to be a violation of the Geneva Conventions. With this being said, the Syrian Government introduced a law in 2012 prohibiting the care of those wounded in protests against the government or those wounded in opposition-occupied areas, under the premise that it is a fight against terrorism.^33^

The danger of potentially working for pro-opposition forces has led to physicians having to operate in underground networks according to an investigation by PHR.^34^ In areas under opposition control, medical facilities are deemed to be abetting the opposition and are hence considered a hostile. Doctors have been forced to work in provisional clinics under poor conditions and lack of equipment but are still relied on as the last haven for wounded soldiers and civilians. PHR has published reports of patients dying secondary to the reasons they had initially come in for, due to the unsanitary and unsterile conditions healthcare workers are forced to operate under.^35^,^36^ One of the cities most affected has been Aleppo since it was taken under opposition control in 2012. Three-quarters of Aleppo’s population has fled the region whilst only a third of hospitals are still operational despite the reduced number of staff– 5% of the region’s doctors have either left or have been killed.^10^

## Humanitarian Aid

Syria has a deep-rooted, rich cultural and religious history boasting six UNESCO World Heritage sites.^37^ A bustling, popular tourist destination, Syria had been ranked 137th in Human Development Index (HDI) by the United Nations Development Programme (UNDP) in 2011- a ranking considering the nation as being in medium development.^38^ HDI takes into consideration three main aspects: a measure of health, level of education and quality of life. Since the start of the conflict, Syria’s HDI and general development had dropped to low with a ranking of 145 worldwide.^38^ The health index alone has dropped Syria in the global ranking from 113th to 174th in the world which stands to be in the bottom decile.^39^ The crisis and drop-in health indicators have caused for intervention from humanitarian and non-governmental organisations worldwide to support the nation’s failing systems.

### Syrian American Medical Society

Syrian health workers were able to cope with the increased demand for provision and their services at the start of the conflict. However, as the war drew on, their services and provision were soon overwhelmed and depleted and so were no longer to provide for the population. Non-governmental organisations and humanitarian relief organisations have had to support Syria’s public health system by assisting with supplies and facilities to those in need. The Syrian American Medical Society (SAMS), had reported that they managed to provide over 3.5 million medical services to the countries Turkey, USA, Greece, Lebanon, Jordan, Bangladesh and Syria with the latter receiving 3.25 million of those medical services in 2017.^40^ This included medicine for chronic conditions, antibiotics for acute and longer presentations of illness and woman health medication.^40^

### World Health Organisation

The World Health Organisation (WHO) has also played an instrumental role in the humanitarian relief of the country; WHO have introduced a health tracking system known as the Health Resources Availability Mapping System (HeRAMS) which identifies how hospitals and primary health centres are functioning and assess their level of functionality via staffing available and if infrastructure is intact. This allows WHO to target and prioritise their resources to specific areas and regions in Syria who are in need the most.^41^ WHO reported that they were able to provide 14 million treatment nationwide whilst supplying 1294 health care facilities with the required supplies and medicines.^17^ Areas under siege are often restricted by the forces in occupation from receiving any type of access as a means of control and cutting off supply lines to the opposition. Nevertheless, WHO managed to negotiate and supply assistance to over 66% of all areas under siege across the country.^17^

### Médecins Sans Frontières

Médecins Sans Frontières (MSF), also known as Doctors without Borders, is a non-governmental organisation which specialises and caters more specifically to the medical needs of a region. However, MSF reports that they have been denied access to government-controlled areas limiting the number of people they can reach to supply relief. Nonetheless, the organisation still managed to reach and complete 372,700 outpatient consolations in 2016 with just over 450 staff.^42^ Despite their work, their facilities have come under attack without regard for their neutrality and impartially to providing medical assistance to all parties in need. In February 2016, several airstrikes hit an MSF-supported hospital in Idlib, killing 25 people and with an 11 further wounded.^43^ An investigation was requested to the International Humanitarian Fact-Finding Commission (IHFFC) to look into the attack which was suspected to have been carried out by government forces and their allies. Reform was needed to protect health care facilities since countless violations against humanitarian law had been committed without repercussion.

The humanitarian aid is not limited to SAMS, MSF, or WHO as mentioned previously but it is clear that they some of the most prominent organisations in helping bridge the health care deficit in the war.

## Violations of Humanitarian Law

It is an old adage that war must be waged with force proportionate to that presented by the opposition. Proportion does not only apply to force: the benefits of going to war must outweigh the price paid. For example, war could be considered ethical if the outcome is that more human life and humanity is saved than would have been if the war had not been waged.

The principle of proportion of force is engraved in international humanitarian law in the form a customary law. Customary laws are in place in humanitarian law to satisfy the voids not fully covered by treaty law such as the Geneva Conventions. The International Court of Justice defines the basis for these laws as “general practice accepted as law”.^44^ Customary laws play a vital role in upholding the justice in international humanitarian laws; despite not all states have signed all treaty law, they are legally bound and obliged to abide by customary law and face action for violating these rules. Concerning proportionality of force in attack, customary law states:

Launching an attack which may be expected to cause incidental loss of civilian life, injury to civilians, damage to civilian objects … would be excessive in relation to the concrete and direct military advantage anticipated, is prohibited.

All parties involved in the conflict so far can be held accountable for violating this customary law as well as several humanitarian laws since the war began. A report substantiated by human rights groups Amnesty International and Human Rights Watch have documented some of the key violations.^45^ All parties have been accused of indiscriminate attacks on civilian areas through the targeting of medical facilities and local shops. ISIL has been accused of indiscriminate attacks in civilian areas in the form of suicide bombings and the prosecuting its civilians following the employment of stringent Islamic law.^45^ One of the most documented violations during the conflict, however, has been the use of chemical weapons in warfare. A UN report stated that the earliest use of chemical weapons during the conflict had been the use of sarin gas killing 1429 people – 426 being children.^46^

A commission was later set up by the United Nations and the Organisation for the Prohibition of Chemical Weapons (OPCW) investigating the perpetrators behind the attacks as well as discerning whether there were any existing stockpiles. Investigations carried out by the UN with referral to the International Court of Justice were blocked by the Russian government as they voted against inquiry at the UN Security Council. The latest chemical attack followed subsequently at Douma, with casualties rising to 70 according to reports.^47^ Both the European Union and other humanitarian organisations called for the immediate referral to the United Nations Security Council to bring the perpetrators to justice.^48^

Organisations such as the Violation Documentation Centre have been set up specifically to document and record all the humanitarian violations committed by pro and opposition forces. These reports are subsequently submitted to the UN Security Council for assessment of violations. However, there have been constant calls for review and reform in policies in place to protect civilians from such atrocities as well as preventing attacks against health care facilities.

## Preventing Attacks

Whilst we have discussed at length the challenges that health care workers face in light of the conflict and operating under attack, it is important that the plans currently in place, as well as proposals in preventing attacks moving forward, are explored.

In the presence of the MSF and ICRC, organisations which the Secretary-General of the UN, Ban Ki-Moon, considered important partners in combating devastation in conflicts, the UN Security Council announced that it would be adopting the Resolution 2286.^49^ The resolution denounces attacks made against health care and humanitarian workers and their facilities, hospitals and other medical services tending to the wounded and sick. The Council also reminded those members present of the importance of upholding and conducting themselves as per the international humanitarian law and the Geneva Conventions.

An inquiry into the “weaponisation of health care in Syria” by Fouad et al,^14^ stated potential policies that could be instated and the relevant parties responsible for enforcing them to ensure that attacks are prevented against the health care sector in times of war.

The report proposed that the UN Security Council Resolution 2286 should be enforced more harshly through more prompt investigation following potential war crimes and following through with prosecution to act as a deterrent. The responsibility to enforce this would fall on all members of the UN including the Security Council itself.

Being able to assess the situation and standing of the health care system as a whole nationwide is imperative in being able to identify which regions may need more protection and assistance than others. Aid organisations have introduced a number of tools to measure the disparity in health services nationwide. WHO has been particularly successful with the HeRAMS service they operate as they have been able to identify facilities with poor availability and functionality. WHO also have plans to release a tool which measures attacks against health care particularly known as the “Monitoring Violence against Health Care” tool which has yet to be implemented.^14^ This would enable them to collect and analyse data to see where the worst affected services are and implement protective strategies to restore functionality and safety to the area.

Humanitarian aid has provided an incredible amount of relief to not only the crisis in Syria, but the ongoing problems in other areas of the Middle East, namely Yemen, and further countries in Africa. Nevertheless, an increase in the number of donors giving to the organisations would aid in the rebuilding of the health sector quicker whilst providing more relief to civilians through the donation of resources and supplies. Conducting a review and reform over the practices of the organisations themselves would also allow them to become more efficient in the allocation of human resources as well as the allocation of supplies. A study in The Lancet revealed that organisations, particularly WHO, could have been more streamlined and efficient in their reporting of violations and allocations of resources, especially across besieged and active conflict lines.^50^

## Methodology

This paper was generated through a literature review looking at relevant studies and writings looking into the attacks on health care workers and facilities in Syria, examining the reasons why they are being targeted as well as ways to prevent it. Health care workers include doctors, nurses, pharmacists, paramedics, ambulance technicians as well as medical students and other health care students. The term health care worker also refers to, volunteer group organisations who employ healthcare professionals like the doctors of MSF, volunteers of White Helmets, etc. Health care facilities refer to hospitals, primary health centres and pharmacies.

As mentioned previously, an “attack” on health in the context of this report is used in line with the WHO interpretation identified in the Syria situation report article.^17^ Through a literature review, information and data had been collected through several search engines including Google Scholar, PubMed, MEDLINE, OVID and searches through Google. I had searched through this engine using an amalgamation of keywords including but not limited to “Syria”, “healthcare”, “health care worker”, “humanitarian aid”, and “volunteer”, “International Humanitarian Law”, “Geneva Convention”.

It was important that the information collected was in a timely manner and from a timeline relevant to that of the conflict. Hence, articles from March 2011–December 2017 were searched for matters directly concerning the war. However, information pertaining to government reports, policies, and legal statements, was not set to a time limit – as long as the information was still relevant and applied to the context. This information was mainly discovered through the following organisation’s websites: “Médecins Sans Frontières”, “WHO”, “ICRC”, “IHL”, “UN”, “UNDP”, etc.. The selection criteria for paper and data used were set to all reputable sources including the organisations listed previously as well as government reports, policies and legal statements.

Collecting data pertaining attacks had been difficult due to discrepancies on occasion between sources as to exact figures. As a result, several sources are provided below in the results section offering an unbiased and inclusive insight into exact figures. Statistics were also derived from non-governmental organisations who should have an unbiased and impartial standpoint which ensures that reporting bias is kept to a minimum. These organisations include the World Health Organisation and MSF.

Much of the discussion of the results seen below has been discussed previously in the report with the following a summary of what had been explored. The strengths and weaknesses of the method will be discussed as well as discussing any unanswered questions.

## Results and Discussion

Through the direct and indiscriminate targeting of healthcare facilities, health care workers have been killed as collateral damage. Table 1 is a collection of data from various non-governmental organisations who endeavour in collecting data which is later collated for either yearly reports from the respective organisations or to report the data to organisations like the United Nations who may refer cases to their Security Council. The years 2012–2014 saw the greatest death tolls amongst all three of the organisations.

**Table 1:** Number of Health Workers Killed in Syria Between 2011–2017

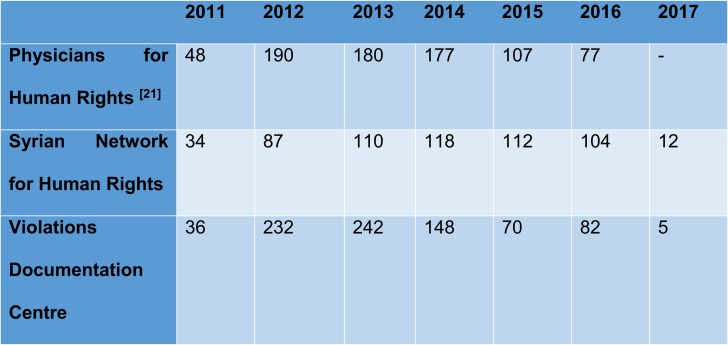

**Notes:** Shows the number of health care workers that have been killed since the start of the Syrian conflict between 2011 and 2017. Adapted from *The Lancet*. 2017;390(10111):2516–2526. Fouad FM, Sparrow A, Tarakji A, et al. Health workers and the weaponisation of health care in Syria: a preliminary inquiry for The Lancet–American University of Beirut Commission on Syria. © 2017 Elsevier Ltd. All rights reserved, with permission from Elsevier.^14^

Due to discrepancies in the figures stated by different organisations, three separate organisations are included to improve accuracy. However, it is still evident that there is a large difference year on year between the different organisations in the figures reported which makes it less accurate and reliable.

**Figure 4:**
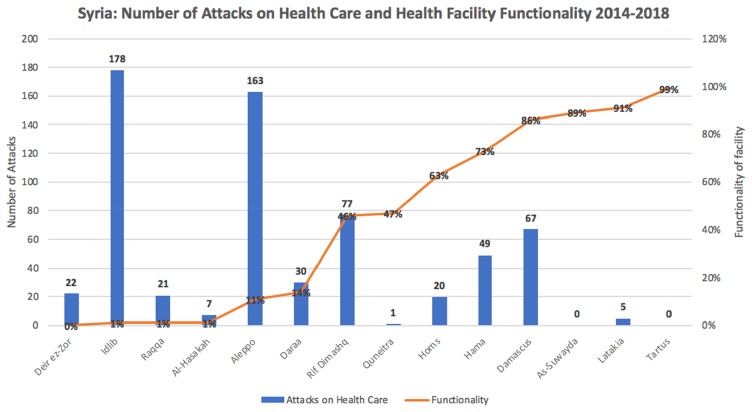
Number of health care facilities attacked and facility functionality between 2014 and 2018. **Notes:** Shows the number of attacks on health care facilities in all Syrian Governorates between 2014 and 2018. It also shows the level of functionality since 2014 for the facilities in question. Adapted from UN and WHO data.^49^,^51^

Figure 4^49^,^51^ shows the profound effect that attacks on facilities have on its long-term functionality. Several facilities are operating at 1% or less functionality then what they should be operating at; Idlib has seen 178 on its services and facilities are currently running at 1%. The stark contrast can be seen on the other end of the spectrum, with Tartus not having experienced any attacks on its provisions in the last four years and is currently operating at 99%. However, As-Suwayda has also been fortunate enough to not have experienced an attack with Quneitra having experienced one but they are operating below that of Tartus with 89% and 47% functionality, respectively.

With the figures extracted from WHO data, it was not made clear exactly what functionality pertained to. There are also geographical differences and differences in control, so a direct attack might not be directly linked to functionality. For example, service provision may already be low since the region is in a rural area, so functionality might be lower than normal.

**Figure 5:**
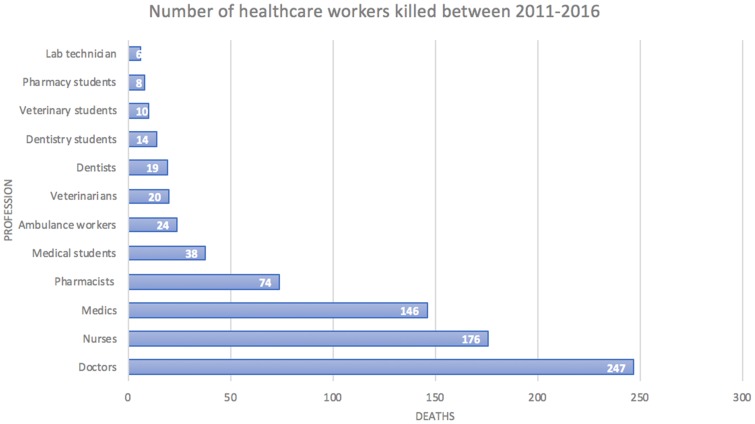
Number of health care workers killed between 2011 and 2016. **Notes:** A chart showing the number of medical personnel killed between 2011 and 2016 broken down by profession. Figure adapted from data from Physicians for Human Rights and data interpreted by Fouad et al.^14^,^21^

Figure 5 presents an estimated 782 medical personnel had been killed during this time with doctors accounting for 32% of total deaths in the five years.^14^,^21^ It is the stark reality of these statistics and the life healthcare professionals live in these conditions which have led to 15,000 of Syria’s 30,000 doctors leaving the country by the end of 2015, a commission in the Lancet recently found.^52^ However, the exact cumulative number of health workers being killed is unknown as the PHR has not released any significant data to continue. PHR has often underestimated the number of deaths amongst medical personnel due to its data collecting methods; the number of deaths is only linked to medical facilities which are protected under IHL. As a result, a large number of deaths amongst makeshift hospitals and unregistered facilities may be missed.

Through the results concluded in this section and earlier in the report, it is evident that the conflict in Syria has had a profound impact on the provision of health care as functionality drops severely along with health indicators. However, much not light has been shed on whether doctors are returning to the country following initially leaving. This is information that the Syrian Medical Syndicate has access to but has not been made public as of, yet which is something that could be explored further. There is also an area of uncertainty in the level of quality of health provision being provided since many of the doctors practising have had to either interrupt their training or have been forced to work in situations that they are not fully trained in due to the urgency for care. This is an area that needs to be explored further as this could have a profound impact on the level/quality of care provided; in turn affecting overall public health.

## Limitations

Collecting data pertaining attacks had been difficult due to discrepancies on occasion between sources as to exact figures. As a result, several sources have been provided in Table 1, offering an unbiased and inclusive insight into exact figures. In addition, t the discrepancies in data reported, statistics were derived from governmental sources and non-governmental organisations who have a reputation for being unbiased and impartial standpoint; this mitigates the chances of reporting bias is kept to a minimum. These organisations included but are not limited to the World Health Organisation and MSF. Another limitation to the study was being restricted to using sources written in English due to the lack of available resources facilitation the translation of Arabic text.

## Conclusion

The struggle that the medical personnel have persevered through during this conflict is testament to the difficulty of operating provisions under the stress of war. More so, it highlights the need for protection from humanitarian violations as health care continues to be targeted as a strategy of war. The report has emphasised how the number of attacks has steadily remained constant throughout the years and nothing seems to be done in bringing perpetrators to justice for violations of International Humanitarian Law. This is made difficult by legislation and nations vetoing referrals to the International Court of Justice and UN Security Council on behalf of other countries. Nonetheless, non-governmental organisations like the Violations Documentation Centre must continue to endeavour to document all violations to corroborate with the UN to continue protecting people’s right to accessing health care. In order for these violators of IHL to be prosecuted, more public attention is needed to shed light on the atrocities being committed; going forward, inquiries like the preliminary carried out by The Lancet – American University of Beirut, will only help in collecting evidence and insight into the weaponisation of health care – ensuring that the health care system and people of Syria obtain the protection and security they warrant.
